# Online Kinematic and Dynamic-State Estimation for Constrained Multibody Systems Based on IMUs

**DOI:** 10.3390/s16030333

**Published:** 2016-03-04

**Authors:** José Luis Torres-Moreno, José Luis Blanco-Claraco, Antonio Giménez-Fernández, Emilio Sanjurjo, Miguel Ángel Naya

**Affiliations:** 1 Department of Engineering, Automatic Control, Robotics and Mechatronics Research Group, University of Almería, Agrifood Campus of International Excellence (ceiA3), CIESOL, Joint Center University of Almería-CIEMAT, Almería 04120, Spain; joseluisblancoc@gmail.com (J.L.B.-C.); agimfer@ual.es (A.G.-F.); 2 Mechanical Engineering Laboratory, University of A Coruña, Escuela Politécnica Superior, Mendizábal s/n, Ferrol 15403, Spain; emilio.sanjurjo@udc.es (E.S.); minaya@udc.es (M.Á.N.)

**Keywords:** kinematics, dynamics of multibody systems, simulation, state estimation, Kalman filter, testbed, inertial measurement units

## Abstract

This article addresses the problems of online estimations of kinematic and dynamic states of a mechanism from a sequence of noisy measurements. In particular, we focus on a planar four-bar linkage equipped with inertial measurement units (IMUs). Firstly, we describe how the position, velocity, and acceleration of all parts of the mechanism can be derived from IMU signals by means of multibody kinematics. Next, we propose the novel idea of integrating the generic multibody dynamic equations into two variants of Kalman filtering, *i.e.*, the extended Kalman filter (EKF) and the unscented Kalman filter (UKF), in a way that enables us to handle closed-loop, constrained mechanisms, whose state space variables are not independent and would normally prevent the direct use of such estimators. The proposal in this work is to apply those estimators over the manifolds of allowed positions and velocities, by means of estimating a subset of independent coordinates only. The proposed techniques are experimentally validated on a testbed equipped with encoders as a means of establishing the ground-truth. Estimators are run online in real-time, a feature not matched by any previous procedure of those reported in the literature on multibody dynamics.

## 1. Introduction

Motion measurement, an expanding field of research, has surged due to the sustained development of new generations of sensors related to motion-capture techniques. In this work, we focus on a new application, tailored to the evaluation of kinematic and dynamical models based on the principles of classical mechanics. Some of these principles were first formulated during the Renaissance, when the subject of Mechanism and Machine Science underwent a remarkable revolution. Although the first teaching specifically dedicated to this subject is attributed to Galileo [1], more than three centuries elapsed before Kinematics became an established discipline after the contributions of mathematicians and mechanical engineers, including the influential figures of Lorenzo Allievi and Ludwig Burmester [2]. The techniques developed were based on either graphical or analytical methods, which, on the one hand, the graph-analytical methods relied on a geometrical approach that lead to a graphical or analytical solving procedure. On the other hand, analytical methods were based on the equations that define the position of each element in a mechanism. Graph-analytical methods are still used today, especially in education. However, they are clearly inefficient when dealing with complex real-world systems such as those employed in the industry or in research. With the aim of overcoming those limitations, new methodologies for the kinematic and dynamic analysis of mechanical systems were developed in the second half of the 20th century, coinciding with the introduction of the first digital computers. Next, we introduce these kinds of modern methods, typically clustered under the name of Multibody Systems Dynamics (MBSD).

A Multibody System (MBS) is defined as a set of rigid or flexible bodies connected to each other by kinematic joints, affected by external and inertia forces which generate reaction forces in these joints as well as a given state of motion with respect to an inertial reference frame [3]. MBSD has been treated as an independent discipline within Mechanics since 1977, when the first specific international symposium (entitled Dynamics of Multibody Systems) was held in Munich (Germany) sponsored by the International Union of Theoretical and Applied Mechanics (IUTAM). Currently, numerous research efforts related to the MBSD focus on achieving more efficient computational algorithms to speed up MBS simulations, on the development of state observers, or even on the use of symbolic manipulation techniques for automatically determining the equations of motion of a particular mechanism or machine.

**Figure 1:**
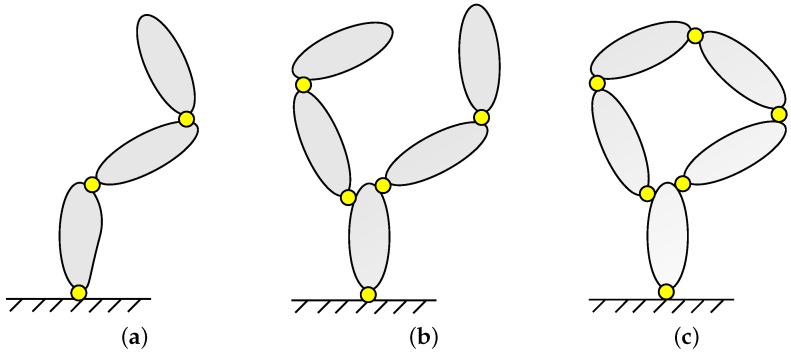
Typical topologies of multibody systems. (**a**) Open-loop topology; (**b**) Tree topology; (**c**) Closed chain.

One of the aspects that most importantly affect the treatment of a MBS is its topology, that is, how its elements are interconnected to each other. Figure 1 illustrates the three main different topologies that a MBS can adopt. The first topology, shown in Figure 1a, corresponds to serial chains or open-loop mechanisms, in which each body is connected to a preceding body and is the basis of the next one, with the exception of the first body (whose preceding body is the ground) and the last one (the end effector or leaf). A typical example of this topology is a robotic arm. Another possibility, sketched in Figure 1b, is the tree structure. This is characterized by the existence of at least one element which acts as base for two or more bodies. These types of mechanisms feature multiple end effectors. The human skeleton can be modeled under this type of topology. Finally, Figure 1c represents a closed-loop topology, which can be seen as a tree where one or more additional connections have been added, establishing closed loops in the topology. This topology is related to constrained multibody systems, and its particularities are exploited in the present work. A classical example within this topology is the four-bar linkage, which is present in numerous applications and machines.

The literature on multibody systems covers a high variety of topics. Among the most theoretically shifted works, we find the development of new formulations [3,4,5], the study of how to introduce flexible bodies and joints [6,7], the analysis of contacts and impacts [8,9] or efficiency improvements [10,11,12]. Other works have focused on practical applications such as vehicle dynamics [13,14,15], robotics [16,17] or biomechanics [18,19]. Some of these works incorporate experiments with real prototypes, aimed at verifying assumptions, evaluating the uncertainty of models, analyzing the influence of tuning certain parameters or even developing new applications.

A particularly challenging aspect of MBS research involves the fact that experimental validation requires the measurement of motion, that can become quite cumbersome. First, we find the limitations of the sensors themselves:

- Accelerometers are suitable for the analysis of vibrations. However, precise measurements of the position and velocity of a body exclusively from their signals is not practical because of the large errors that result from integrating accelerations.
- Encoders ensure noise-free measurements of the angular position of an axle in a given rotatory motion. It is relatively easy to determine angular velocity by differentiating such signals with respect to time. The main disadvantage of these sensors involves their physical placement. It is not always possible to gain access to the part of a machine where the sensor should be positioned. This is especially important in biomechanics. Furthermore, encoders are typically rather expensive and bulky devices.
- Computer vision systems: Motion capture based on computer vision is increasing becoming widespread in recent years due to its suitability for remote, non-contact sensing. However, the systems invariably require an accurate calibration process [20], and expensive imaging sensors may be needed if the system under study moves at high speed. A major drawback of computer vision is the line-of-sight restriction.
- Inertial Measurement Units (IMUs): These sensors arise as useful alternative. Typically the combination of gyroscopes together with accelerometers and magnetometers provides high-rate, accurate information on three-dimensional motion. IMUs can offer greater placement flexibility than encoders can, and also provide more precise measurement of angular accelerations because the gyroscope output needs to be differentiated only once.

Another important issue regarding the study of motion measurement is that predictions are made solely on system models —e.g., from a CAD design—and simulated via MBS dynamics, leading to unavoidable errors. Under real-world conditions, mechanical systems are characterized by having a large number of parameters which lead to uncertainties and the need of more complex mathematical models. When dealing with machines, it is not always possible to have exact information on all their components. Kinematic errors are inherent to misalignments or geometrical deviations. Unknown external forces, masses, inertias or friction coefficients also induce important uncertainties in the dynamical state. Thus, a novel approach for the simultaneous estimation of the kinematic state and the unknown forces affecting to MBSs composed of rigid links has been presented in [21] with promising results. When the human body is treated as a mechanical system (*i.e.*, in biomechanics), uncertainties become even more critical [22]. These systems are characterized by open-loop topologies in which the joints between the different elements are far from the ideal revolute, prismatic or cylindrical pairs. Instead, each different joint of each human corresponds a specific kinematic behavior. This phenomenon, in combination with the difficulties in the modeling of the actuators (muscles), raises an attractive challenge for the problem of measuring and modeling the motion of complex mechanical systems.

Several works involving inertial sensors for motion analysis of robotics actuators and human limbs can be found in the literature [23,24,25]. For example, a novel approach has been proposed [23] for estimating the flexing and extending angle of human limbs by identifying the joint axes and positions via minimization algorithms, considering integrations of angular rates from gyroscopes and drift-free angle estimation from accelerometers. The complementary filter is employed as a sensor fusion tool, but without using any model information as proposed in the present work. This methodology was presented as suitable for real-time contexts, but real-time capability was not tested experimentally. Elsewhere [24], a similar challenge is covered in which the inertial posture tracking is undertaken using complementary filtering and Kalman filtering. Since each device must process the sensor data locally due to limitations on the transmission of data, special attention is given to the algorithm’s computational cost. The algorithms based on Kalman filtering arise as much less accurate in comparison with those using complementary filtering, due to the method’s dependency on realistic process modeling. In addition, the computational cost is greater. These inconveniences are overcome in our work by the use of more accurate models and real-time capable execution code, as discussed in Section 5. Unlike the aforementioned studies in which the ground-truth consists of expensive and cumbersome optical tracking systems, the present work employs encoder signals to validate our approach, thereby simplifying the experimental tasks. Furthermore, it should be stressed that none of these previous works handle closed-loop topologies, in which it is difficult to integrate the corrections of the observer and conserve the properties of rigid solids such as constant lengths. A distinguishing feature of our approach is that we address these specific constraints.

Due to the better knowledge of the dimensional and inertial characteristics of robotic members, as well as the joint behavior, in a former study [26] the estimation of the center of mass of a biped robot is complemented with a Kalman filter involving the system equations of motion. The robot legs are simplified to be treated as an inverted pendulum, leading to an open-loop topology multibody system. This approach leads to the use of expensive sensors such as force/torque sensors and joint sensors in addition to the IMU. In contrast, our approach focuses on the idea of coupling techniques of multibody systems dynamics and state observers. We have exploited the characteristic of the multibody systems that allow the use of dependent coordinates on their modelization together with the dynamic equations, imposing certain restrictions between the coordinates that define the system (constraint equations). This allows the dynamics of complex systems to be addressed, with open or closed chains, in a more general way, ensuring that the conditions of the system are satisfied. The simultaneous resolution of the dynamics and constraint equations has its own techniques (see the references [3,5,6]). Among the papers found in the literature dealing with state observers under this framework (on which the present paper focuses), only those two studies, [14,27], include experiments based on real systems. The former considers position-level data collected by real sensors, whereas the latter uses simulated signals. However, none of the references found deals with inputs that come from sensors at velocity level or that perform the online estimation simultaneously with the real system. This is the main motivation for this work, which concentrates on the use of IMUs to determine the kinematic and dynamic state of MBSs.

The rest of this work is organized as follows. We firstly present the theoretical background on kinematics from the perspective of the MBSD approach in Section 2. Next, Section 3 introduces the modeling of the particular four-bar linkage employed as a testbed, the particularities on how to map sensor readings to theoretical predictions and the implementation of two variants of the Kalman filter as a way to predict its dynamical state. We have selected this mechanism for the sake of simplicity, although the filter formulations proposed in this section are expressed in three-dimensional space. Afterwards, Section 4 describes the experimental setup which makes up the physical mechanism, its sensors, and the hardware architecture developed. Finally, we present the experimental results in Section 5 and draw some conclusions.

## 2. Theoretical Background

Modeling is the first activity needed to carry out a simulation under the MBSD approach. As mentioned above, this stage was not present in the same form as it was in traditional Mechanical Engineering methods—*i.e.*, graphical and analytical methods—derived from a direct application of the classical mechanics laws in the field of Kinematics and Dynamics of Machines. In turn, modeling a MBS consists of identifying the parts of a given mechanical system and how to express them mathematically as an appropriate set of variables. Thus, concepts such as rigid body, kinematic joint or velocity field become a set of vectors and matrices, suitable for computer simulations.

Hence, it is advisable to identify the parameters which univocally define the position, velocity, and acceleration of all the elements of the system at each time step. This information is provided through the generalized coordinates of the system. The strategic and smart selection of these coordinates along the different bodies of a multibody system is one of the pivotal stages of the modeling process.

Generalized coordinates are grouped in a vector **q**, adopting any arbitrary value which satisfies the constraint equations **Φ**. The position of the mechanism is thus assumed to be compatible with its kinematic chain. Expressed in algebraic terms, this assumptions leads to a set of non-linear constraint equations:

(1) Φ(q,t)=0

whose resolution by numerical methods is referred to as the position problem [3]. Similarly, the constraint equations must be accomplished for the system velocities. The time derivative of Equation (1) states the velocity problem, characterized by the following expression:

(2) $$\Phi_{q}(q,t)\;\dot{q}=0$$

where **Φ_q_** represents the Jacobian matrix of the constraint equations and the velocity-dependent constraints have been neglected. Similarly, the second time derivative of the constraint equations leads to the acceleration problem:

(3) $$\Phi_{q}(q,t)\;\ddot{q}+{\dot{\Phi }}_{q}(q,\dot{q},t)\;\dot{q}=0$$

Regarding the definition of coordinates, the main decision is whether to choose dependent or independent coordinates, a classification related to the way in which they define the position of the different bodies. Independent coordinates are suitable for systems defined by open-loop topologies. Thus, a relative coordinate may be employed in each joint. On the other hand, dependent coordinates arise as a more convenient alternative for closed-loop topologies. Several types of coordinates can be used as dependent coordinates. The most popular are reference-point coordinates, relative coordinates, and natural coordinates. Natural coordinates were proposed elsewhere [28]. Some of their advantages involve increasing the computational efficiency of simulations, simplifying the modeling process and decreasing the complexity of the dynamic problems.

**Figure 2:**
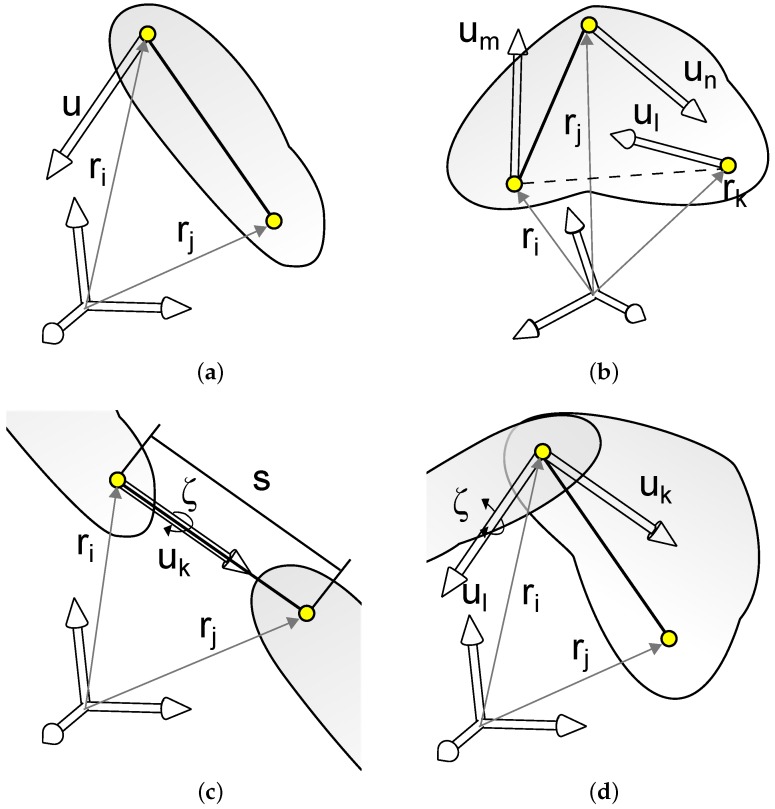
Modeling rigid bodies and kinematic constraints under the MBSD approach. (**a**) Two points and a unitary vector; (**b**) An algebraic basis; (**c**) Cylindrical joint; (**d**) Revolute pair.

When using natural coordinates, the kinematic state of each body in a multibody system is defined by a set of variables comprising (a) coordinates of points and (b) vectors. This may be achieved in different ways, such as using three non-aligned points or two points plus a unitary vector. The latter option corresponds to the sketch in Figure 2a. For complex bodies, it is advisable to form an algebraic basis. Figure 2b shows a body defined by the basis formed through the points represented by $r_{i}$ and $r_{j}$ and the vectors $u_{m}$ and $u_{n}$. Any additional point or vector is introduced as linear combination of such a basis.

Generally, each body leads to *m* constraint equations, resulting from the difference between the number of coordinates employed on its modelization (*n*) and its number of degrees of freedom (d.o.f.). By assuming ideal rigid bodies, Equation (4a) ensures the corresponding condition of constant length. Moreover, Equation (4b) states a generic vector **u** to be a unitary vector. Finally, Equation (4c) allows a constant angle to be determined between two arbitrary vectors **u** and $r_{ij}$

(4a) $$r_{i,j}\cdot r_{i,j}-L_{i,j}^{2}=0$$

(4b) u·u−1=0

(4c) $$r_{i,j}\cdot u-L_{ij}\cos\phi =0$$

Also, joint constraints an be used. It is a good practice to share points and vectors between the bodies forming the kinematic pair, which is common in revolute joints. Additionally, cylindrical joints may be expressed in the following form:

(5) $$u_{k}\wedge r_{i,j}=0$$

where the alignment $u_{k}\wedge r_{i,j}$ is imposed by means of a constant-cross-product condition. Figure 2c represents a cylindrical constraint involving the points *i* and *j* releasing only one d.o.f. corresponding to the relative rotation between the two bodies along $u_{k}$, represented by *ζ*. The distance between them, *s*, may be also included as a relative coordinate into the model by means of the following relative coordinate constraint equation:

(6) $$\left(r_{j}-r_{i}\right)\cdot \;\left(r_{j}-r_{i}\right)-s^{2}=0$$

Finally, Figure 2d represents an angular relative coordinate which allows the angle *ζ* to be introduced into the model. The corresponding constraint equation takes the form:

(7) $$u_{k}\cdot \frac{\left(r_{j}-r_{i}\right)}{|r_{j}-r_{i}|}-\cos\zeta =0$$

As a particular case of the three-dimensional space, the natural coordinates in the plane are composed of the Cartesian coordinates of some points of each solid placed in the joints. The following rules should be taken into account when modeling a planar mechanism by using natural coordinates [3]:

- Each solid must contain, at least, one point and one vector, or two points. If this condition is not met, it is not possible to determine the orientation of such body.
- For the revolute joints to be considered implicitly, it is strongly recommended that a point be placed at each joint.
- In the case of prismatic joints, at least three aligned points are necessary: two for defining the axis of the displacement and one more for the slider. Alternatively, these kinds of joints can be also defined by using two points and a vector.
- As many additional points can be used as desired.

## 3. Methodology

### 3.1. Kinematic Modeling of the Experimental Testbed

**Figure 3:**
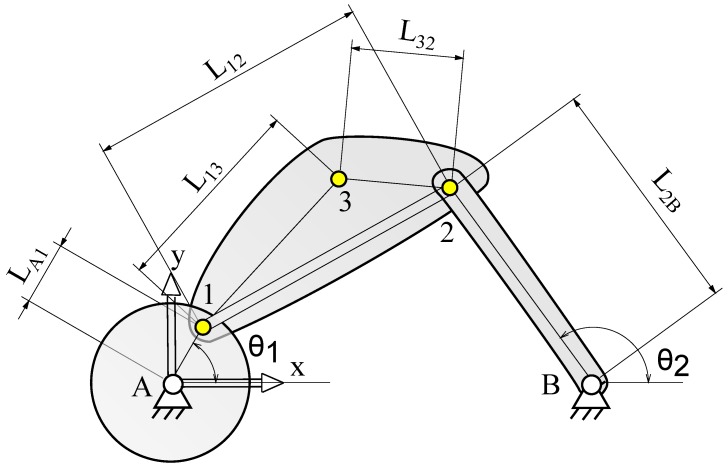
Schematic of the attached problem.

The problem under study in this paper consists of a planar four-bar mechanism that includes an additional point in the coupler link, as sketched in Figure 3. The crank is a disk of radius *r* and mass $m_{A1}$. In terms of Kinematics, this element is equivalent to a link of length $L_{A1}$. The coupler link is a thin bar of length $L_{12}$. Attached to this bar, a bracket allows the first of the two IMUs to be placed in a strategic position referred to as a coupler point, rendering the kinematic problem more challenging. As depicted in Figure 3, the coupler link together with the bracket supporting the first IMU form a unique body modeled by three points having a mass $m_{12}$. Finally, the rocker link is another bar of length $L_{2B}$ and mass $m_{2B}$. All the pairs are assumed to be ideal revolute joints.

The vector of generalized coordinates is $q={\left[x_{1}\;y_{1}\;x_{2}\;y_{2}\;\theta_{1}\;x_{3}\;y_{3}\;\theta_{2}\right]}^{⊤}$. This is a clear example of redundant coordinates: the first subset ${\left[x_{1}\;y_{1}\;x_{2}\;y_{2}\right]}^{⊤}$ is the minimal set of natural coordinates that guarantee the unequivocal definition of the mechanism position. The other coordinates are included within the model for the following purposes: angle $\theta_{1}$ allows the proper introduction of the motor actuation into the model. This will be the coordinate considered as the degree of freedom of the device. Coordinates $x_{3}$, $y_{3}$ correspond to the coupler point. These coordinates may be obtained as linear combination in the basis formed by the coupler link. However, their introduction allows a seamless integration of information from the first IMU sensor. Finally, the addition of $\theta_{2}$ is based on the same criterion, now considering the angular measurements of the rocker link from both the second IMU and the encoder. Since the number of coordinates (*n*) is 8, and the number of d.o.f. is 1, we need m=n−1=7 constraint equations. Thus, the constraint equations which form the vector Φ(q,t)=0 are:

(8a) $${\left(x_{1}-x_{A}\right)}^{2}+{\left(y_{1}-y_{A}\right)}^{2}-L_{A1}^{2}=0$$

(8b) $${\left(x_{2}-x_{1}\right)}^{2}+{\left(y_{2}-y_{1}\right)}^{2}-L_{12}^{2}=0$$

(8c) $${\left(x_{2}-x_{B}\right)}^{2}+{\left(y_{2}-y_{B}\right)}^{2}-L_{2B}^{2}=0$$

(8d) $$\left(x_{1}-x_{A}\right)-L_{A1}\;\cos\theta_{1}=0$$

(8e) $$\left(x_{2}-x_{1}\right)\left(x_{3}-x_{1}\right)+\left(y_{2}-y_{1}\right)\left(y_{3}-y_{1}\right)-L_{12}\;L_{13}\;\cos∠213=0$$

(8f) $${\left(x_{3}-x_{1}\right)}^{2}+{\left(y_{3}-y_{1}\right)}^{2}-L_{13}^{2}=0$$

(8g) $$\left(x_{2}-x_{B}\right)-L_{2B}\cdot \cos\theta_{2}=0$$

where the Equation (8a–c) represent the condition of rigid body of the disk and the two bars, respectively, Equation (8d,g) define the relative coordinates $\theta_{1}$ and $\theta_{2}$ respectively, and Equation (8e,f) with regard to the constant angle between the segments $\bar{12}$ and $\bar{13}$ as well as the constant length from the coupler point 3 to point 1.

Most of the kinematic variables of the model are readily accessible as natural and relative coordinates in the dynamic state vector $\left\{q,\dot{q}\right\}$. However, determining the angular velocity of floating links requires further transformations. It can be shown that the following expression, which will be used to simulate gyroscope sensors, allows us obtaining the angular velocity of a rigid link between two arbitrary points *i* and *j*:

(9) $$\omega_{ij}=\frac{\left({\dot{x}}_{j}-{\dot{x}}_{i}\right)\cdot \left(y_{i}-y_{j}\right)+\left({\dot{y}}_{j}-{\dot{y}}_{i}\right)\cdot \left(x_{j}-x_{i}\right)}{L_{ij}^{2}}$$

Once the system is modeled, the procedure to perform a kinematic simulation is as shown in Algorithm 1. This model will be used in the experiments described in Section 5.1, with the aim of demonstrating how positions, velocities, and accelerations of each element of the testbed may be predicted from known values of its degree of freedom. In our case, this value is $\theta_{1}\in q$, and hence the index of the d.o.f coordinate, $i_{dof}$ in the algorithm, would be 5, according to the list of coordinates described in Section 3. Note that the right-hand-side vector of the velocity problem (${\mathrm{rhs}}_{v}$) is formed by incorporating the known velocity of the variable treated as d.o.f.


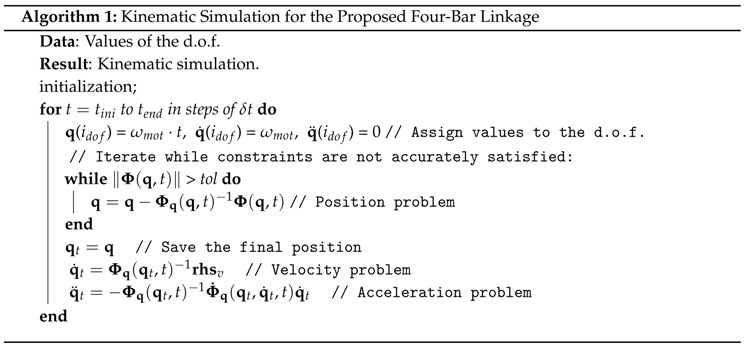


### 3.2. Modelization of Virtual Sensors

Above, we discussed how to solve the position, velocity, and acceleration problems, allowing us to determine the kinematic variables of interest, in all cases referring to a global coordinate framework. However, when dealing with accelerations from the inertial units, some transformation needs to be taken into account so as to be able to compare them with the results predicted by kinematic simulations. Thus, the acceleration of the coupler point measured by the first IMU and referred to the global-reference system of the linkage is calculated as:

(10) $$a=A\cdot (a_{\mathrm{IMU}}+A^{⊤}\cdot g)$$

where **a** represents the acceleration denoted in global coordinates of the point 3, which can be decomposed into its Cartesian components ${\ddot{x}}_{3}^{*}$ and ${\ddot{y}}_{3}^{*}$. The rotation matrix **A** allows to transform the body local coordinates to global coordinates and its columns are the basis of the local system expressed as global coordinates by means of an angle *φ* which stands for the planar orientation of the bracket containing the IMU at each moment. The term $a_{\mathrm{IMU}}$ contains the accelerations *acc_x_* and *acc_z_* read by the IMU. Finally the vector **g** is related to the gravity acceleration. This term reflects the fact that the effects of gravity must be subtracted, since inertial sensors measure the proper accelerations as defined in relativistic mechanics [29], whereas in classical mechanics we always handle coordinate acceleration as defined in an inertial, global-reference framework. Table 1 shows a summary of the notation used to clearly address either the measured signals from sensors or the corresponding values from the kinematic simulation.

Regarding how to track the position of the points of interest, the coordinates of points 1–3 can be estimated in a more straightforward manner from the kinematic chain geometry and the IMUs-provided angles *φ* and *ψ*, as follows:

(11a) $$x_{1}^{*}=x_{2}^{*}-L_{12}\cos\phi$$

(11b) $$y_{1}^{*}=y_{2}^{*}-L_{12}\sin\phi$$

(11c) $$x_{2}^{*}=L_{AB}+L_{2B}\cos\psi$$

(11d) $$y_{2}^{*}=L_{2B}\sin\psi$$

(11e) $$x_{3}^{*}=x_{1}^{*}+L_{13}\cos\left(\phi +∠123\right)$$

(11f) $$y_{3}^{*}=y_{1}^{*}+L_{13}\sin\left(\phi +∠123\right)$$

Note that these coordinates are the predictions based solely on IMU data. They are to be compared to the result of the kinematic simulation, *i.e.*, the position problem in Algorithm 1, considering as input the angular position of the crank measured with the optical encoder.

### 3.3. Dynamical Modeling and State Estimation

The kinematic modeling approach followed in the foregoing sections constitutes a powerful tool for the dynamic analysis of the mechanism under study, since solving the equations of motion leads to a vector of generalized coordinates which satisfies the constraint equations. Hence, the positions and velocities are solved accordingly in order to ensure the fulfillment of the constraints. Other approaches such as analytical techniques—e.g., Raven’s method [30]—may address less complex solutions for solving the linkage kinematic problem. However, when dealing with dynamic simulations, these methods present serious limitations. The MBSD approach in turn proves more suitable in this situation, as mentioned above. Thus, when the modeling process presented in Section 3.1 is taken into account, its application to the attached problem is straightforward. In the following subsections, we introduce a procedure for performing state estimations for MBSs based on nonlinear filters, namely, the Discrete Extended Kalman Filter (DEKF), and the Unscented Kalman Filter (UKF) (see Table 2 for a summary of the notation employed from here on).

#### 3.3.1. Discrete Extended Kalman Filter (DEKF)

This formulation is the discrete version of the Kalman Filter, comprising two separated stages: state transition—also called prediction—and state update. The former relies on the transition model of the system defined by equations of motions and constraint equations, while the latter includes the information from sensors, or observations. Each stage comprises differentiated equations for updating the state vector and the covariance matrix.

This formulation has been described in previous works [31], but will be reproduced here for the convenience of reader. The main idea under this formulation is to adapt the multibody equations in order to fit the Kalman filter structure. In its most basic form, the dynamics of a multibody system is described by the constrained Lagrangian equations:

(12) $$\left\{\begin{array}{ccc} M\ddot{q}+{\Phi_{q}}^{⊤}\lambda & = & Q\\ \Phi & = & 0 \end{array}\right.$$

where **M** represents the mechanism’s mass matrix, **λ** the Lagrange’s multiplier and **Q** the vector of generalized forces. The multibody formalism employed is the R-matrix formulation [3], aimed at obtaining an ODE with dimension *g* equal to the number of degrees of freedom. This formalism is based on the identity $\dot{q}=R\dot{z}$, which relates dependent and independent velocities by means of the **R** matrix. Accelerations can be then expressed as follows:

(13) $$\ddot{q}=R\ddot{z}+\dot{R}\dot{z}$$

Going back to Equation (12), premultiplying by the transpose of R, and bearing in mind that $\Phi_{q}R=0$,

(14) $$\ddot{z}={\left(R^{⊤}MR\right)}^{-1}\left[R^{⊤}\left(Q-M\dot{R}\dot{z}\right)\right]={\bar{M}}^{-1}\bar{Q}$$

where ${\bar{M}}^{-1}$ is the corrected mass matrix and $\bar{Q}$ is the corrected vector of generalized forces. If now the filter state is defined as the vector $x^{⊤}=\left\{z^{⊤},\dot{z}{}^{⊤}\right\}$, it turns out that:

(15) $$\left\{\begin{array}{c} \dot{z}\\ \ddot{z} \end{array}\right\}=\left\{\begin{array}{c} \dot{z}\\ {\bar{M}}^{-1}\bar{Q} \end{array}\right\}\;\Rightarrow \;\dot{x}=f(x)$$

Starting with the prediction stage, the EKF equations in their most generic form are:

(16a) $${\hat{x}}_{k}^{-}=f({\hat{x}}_{k-1}^{+})$$

(16b) $$P_{k}^{-}={f_{x}}_{k-1}P_{k-1}^{+}{f_{x}}_{k-1}^{⊤}+\Sigma_{k-1}^{P}$$

where f(·) stands for the transition model of the system. By considering now the state vector of a MBS estimator in independent coordinates, ${\hat{x}}^{⊤}=\left\{{\hat{z}}^{⊤},\hat{\dot{z}}{}^{⊤}\right\}$, and assuming the usage of the Euler method for numerical integration with time step Δt, we can put the integrator in a form that fits that required by the EKF transition function f(·):

(17) $${\hat{x}}_{k}^{-}=f({\hat{x}}_{k-1}^{+})\;⟶\left[\begin{array}{c} {\hat{z}}_{k}\\ {\hat{\dot{z}}}_{k} \end{array}\right]=\left[\begin{array}{c} {\hat{z}}_{k-1}+\Delta t{\hat{\dot{z}}}_{k-1}\\ {\hat{\dot{z}}}_{k-1}+\Delta t{\ddot{z}}_{k-1} \end{array}\right]$$

Here, the only unknown term is the acceleration vector ${\ddot{z}}_{k-1}$ for the previous time step, which must be computed by solving the multibody equations of motion as in Equation (14). Thus, it follows that the transition model Jacobian $f_{x}$ has a fairly simple structure:

(18) $$f_{x}\equiv \frac{\partial f}{\partial \hat{x}}=\frac{\partial }{\partial \{\hat{z},\hat{\dot{z}}\}}\left[\begin{array}{c} \hat{z}+\Delta t\hat{\dot{z}}\\ \hat{\dot{z}}+\Delta t\ddot{z} \end{array}\right]=\left[\begin{array}{cc} I_{g} & \Delta tI_{g}\\ 0_{g\times g} & I_{g} \end{array}\right]$$

The $\Sigma_{k-1}^{P}$ covariance matrix appearing in Equation (16) stands for the additional uncertainty of the new state ${\hat{x}}_{k}$, physically attributable to unmodeled forces and errors in the parameterization of the mechanism (e.g., lengths of bars, inertia values, *etc.*). Under the assumption of independent and identically distributed Gaussian noise for each independent coordinate, its structure becomes:

(19) $$\Sigma_{k-1}^{P}=\left[\begin{array}{cc} \sigma_{\hat{z}}^{2}I_{g} & 0_{g\times g}\\ 0_{g\times g} & \sigma_{\hat{\dot{z}}}^{2}I_{g} \end{array}\right]$$

with the parameters $\sigma_{\hat{z}}$ and $\sigma_{\hat{\dot{z}}}$ specifying the standard deviations of the assumed noise in position and velocities, respectively.

The second stage of the DEKF method, the *update*, incorporates the sensor readings to improve the estimate:

(20a) $${\tilde{y}}_{k}=o_{k}-h({\hat{x}}_{k}^{-})$$

(20b) $$S_{k}={h_{x}}_{k}P_{k}^{-}{h_{x}}_{k}^{⊤}+\Sigma_{k}^{S}$$

(20c) $$K_{k}=P_{k}^{-}{h_{x}}_{k}^{⊤}S_{k}^{-1}$$

(20d) $${\hat{x}}_{k}^{+}={\hat{x}}_{k}^{-}+K_{k}{\tilde{y}}_{k}$$

(20e) $$P_{k}^{+}=\left(I_{g}-K_{k}{h_{x}}_{k}\right)P_{k}^{-}$$

where h(·) stands for the observation model of the system, such that ${\tilde{y}}_{k}$ in Equation (20a) is clearly the error or mismatch (often called innovation) between the expected sensor readings and their actual values ($o_{k}$). The covariance matrix $S_{k}$ in Equation (20b), or innovation covariance, represents the uncertainty in the system state projected via the sensor function (${h_{x}}_{k}P_{k}^{-}{h_{x}}_{k}^{⊤}$) plus an additional additive Gaussian noise originated in the sensor itself ($\Sigma_{k}^{S}$). Small values of $S_{k}$ mean that the observation introduces useful information to constrain the estimation of the system state. By evaluating the temporary term known as Kalman gain ($K_{k}$), we can update the estimate mean and covariance, in Equation (20d,e), respectively. These values are then used as the input to the next iteration of this iterative filter in the next time step.

#### 3.3.2. Unscented Kalman Filter (UKF)

The Unscented Kalman Filter (UKF) [32] comes as an evolution of the family of Kalman filters that is better suited to cope with strong nonlinearities in the transition and observation models. Comprising the same prediction and update stages than DEKF, the distinguishing feature of UKF is the avoidance of the first-order Taylor approximation in the propagation of Gaussian random variables through the transition and observation functions. Instead, a set of samples are deterministically chosen from the Gaussian distributions, and transformed via the corresponding function. Then those samples in the transformed space are converted back into a parametric distribution, *i.e*., they are used to compute the mean and covariance of the corresponding Gaussian. As shown in [32], this approach captures the correct posterior mean and covariance up to the third order of a Taylor series expansion, in contrast to the first order of DEKF and most other methods. Additionally, this procedure avoids the requirement of developing the Jacobian matrices of the transition and observation functions. In turn, its computational cost is in general higher than for the simpler methods.

For the attached problem, the state vector of UKF is composed of the independent coordinates and their velocities, *i.e.*, ${\hat{x}}^{⊤}=\left\{{\hat{z}}^{⊤},\hat{\dot{z}}{}^{⊤}\right\}$. As mentioned above, each filter iteration bears the same two steps as DEKF, so that only the differences will be highlighted here. Denoting the dimensionality of the state space $|\hat{x}|$ as *L*, a total of 2L+1 deterministic samples (or sigma points) $\chi_{i}$ with i=0,…,2L are generated from the mean ${\hat{x}}_{k-1}^{+}$ and covariance $P_{k-1}^{+}$, each with a different weight $W_{i}$. Then, the samples are transformed with a forward Euler transition function identical to that of previous filters, and the predicted mean ${\hat{x}}_{k}^{-}$ and covariance $P_{k}^{-}$ are estimated from them. A similar process applies to the propagation of the uncertainty in observations, taking into account both the uncertainty in the system state and the sensor noise (referring to the two terms in the innovation covariance of DEKF above). The reader is referred to the original work [32] for the filter equations, not reproduced here for the sake of brevity.

## 4. Experimental Setup

**Figure 4:**
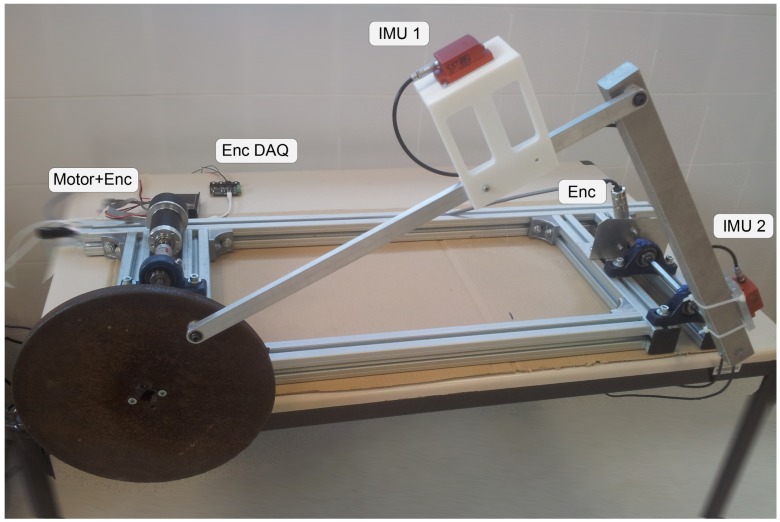
Picture of the prototype employed as testbed for the present work. The two inertial measurement unit (IMU) sensors are clearly visible on the top of the coupler link and at the right hand side of the rocker.

The real linkage for testing the kinematic-based estimation methods based on inertial units is depicted in Figure 4. In the previous section, we explained the kinematics modeling and simulation of this mechanism, and below the real prototype is described in detail. The first body (crank) is a high-inertia disk made of iron weighting $m_{A1}$=11.10 kg. The distance from the center and the pin to the coupler bar is $L_{A1}=0.120$ m. This disk is connected to an electric motor (Maxon EC-Motor), comprising a reduction gear and a quadrature encoder with a resolution of 1024 pulses per revolution (ppr), which ensures a precise control of the crank angular speed. In the kinematic tests, the speed has been fixed to 1.8190 rad s${}^{-1}$, which has been experimentally measured to be quite stable (standard deviation of 0.0042 rad s${}^{-1}$). This speed is achieved when the electric motor is working at 2700 rpm. The coupler consists of a slim bar built in aluminum where the first IMU, a 6 axes *XSens MTi-300*, is placed by means a 3D printed bracket. The length of this bar is $L_{12}=0.540$ m and the distances from the IMU to its pins are $L_{13}=0.384$ m and $L_{32}=0.212$ m, respectively. The overall weight of the bar and bracket is of $m_{12}=0.40$ kg. Finally, the rocker consists of a solid rod of $L_{2B}=0.455$ m and $m_{2B}=1.23$ kg made of aluminum and incorporating another identical IMU. At the rotation axis of this link is placed a SICK DFS61 optical quadrature encoder with a resolution of 10,000 ppr. Both crank and rocker parallel shafts are supported by ball bearings fixed to the chassis, separated by a distance of $\bar{AB}=0.8$ m.

The information of the four sensors (*i.e.*, two IMUs and two optical encoders) are logged in a host computer running a software architecture called OpenMORA [33]. This architecture, developed and co-maintained by the authors of this work, has been successfully used on more complex systems such as electric cars and autonomous robots [34,35].

The inertial units are directly connected to the host PC via USB. Their purpose is to measure the angular position and velocity of both the coupler and rocker links, along with its three-dimensional proper acceleration in a local (moving) frame of reference. Data from both encoders are acquired by means of a Phidgets Encoder HighSpeed 4-Input board which communicates with the host computer via USB. The software architecture uses a publication/subscription pattern, in which several independent processes, also called modules, are executed in parallel. For this experiment, two different modules operating at a frequency of 100 Hz manage the signals from the IMUs and the encoders independently.

For the case of the dynamic tests, some modifications are made. First, the motor in the crank is removed, and an optical encoder is placed instead. This modification leads to a multibody dynamics problem in which the gravity is the only external force. Moreover, significant modifications of the software architecture are undertaken since the time-consuming nature of the calculations required to solve the equations of motion stated in Equation (14). A monolithic application is developed to incorporate the state observer algorithms instead of the modular and general-purpose software architecture employed for the kinematic experiments. This application is based on three main threads. The core is the thread executing the filter with a step time of 1 ms, which read the information from the IMUs through the second thread each 10 *ms*. These observations are processed by the estimation function in order to perform the estimation. The third thread is based on the Phidgets API, which consists of a callback that the main loop uses to compute the crank-angular position and hence the dynamical simulation ground-truth. In addition, other threads are executed in parallel for performing auxiliary tasks such as rendering or data logging.

## 5. Results and Discussion

### 5.1. KinematicS-Based Estimation

In this experiment, the crank of the four-link mechanism was driven by an electric motor as it rotated at a constant speed, as described above. Our goal in this section is two-fold: (i) to verify experimentally whether the predictions from multibody kinematics led to accurate predictions of IMU readings; and (ii) compare the IMU-only dead reckoning trajectories with those computed via Algorithm 1.

**Figure 5:**
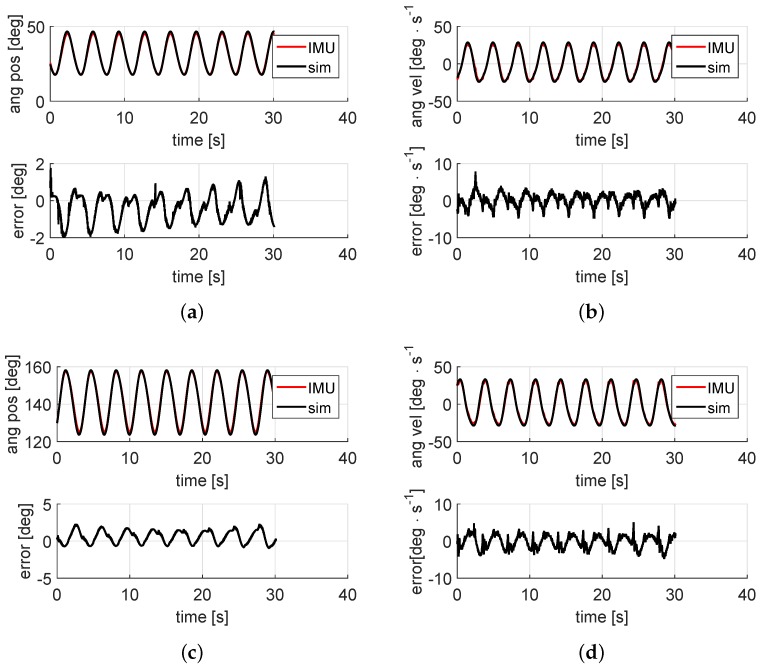
Some results from the experimental validation. In all plots, real data from sensors are superposed over the results from kinematics-based estimations. The left-hand figures show the angular positions of the coupler and rocker links and their estimation errors, whereas their corresponding angular velocities are shown in the right-hand plots. (**a**) Coupler link angular position; (**b**) Coupler link angular velocity; (**c**) Rocker link angular position; (**d**) Rocker link angular velocity.

The theoretical constant speed provided by the motor is the input of the kinematic model. Once the value of the d.o.f. is defined over time, the calculation of the angular position of both the coupler and rocker links is straightforward. Figure 5a,c represents such results, which also illustrate how close the theoretical predictions are to IMU-based measurements: the estimated orientation of the coupler link is shown in Figure 5a, as well as the estimation error, which represents the difference between the predicted and the actual measurements. The root-mean-square error (RMSE) of this estimation is 0.82 deg. Similar results are shown in Figure 5c for the rocker link, where the RMSE is 1.00 deg. Clearly, all results compared match satisfactorily, though with minor discrepancies possibly caused by inaccuracies in the physical parameters (e.g., backlash or angular misalignments) used in the kinematic multibody simulation.

The same procedure is carried out for angular velocities. The angular velocities of the coupler and the rocker links as well as the estimation errors are depicted in the subfigures at the right-hand column of Figure 5. The coupler link angular velocity is shown in Figure 5b, where the data measured by the first inertial unit along with the those from the simulation are represented. Similar results for the rocker link angular speed are depicted in Figure 5d. As can be seen, maximum errors do not exceed 5 $\deg\cdot s^{-1}$, roughly 12% of the link maximum angular speed. The RMSE values are 1.75 $\deg\cdot s^{-1}$ and 1.79 $\deg\cdot s^{-1}$ respectively.

**Figure 6:**
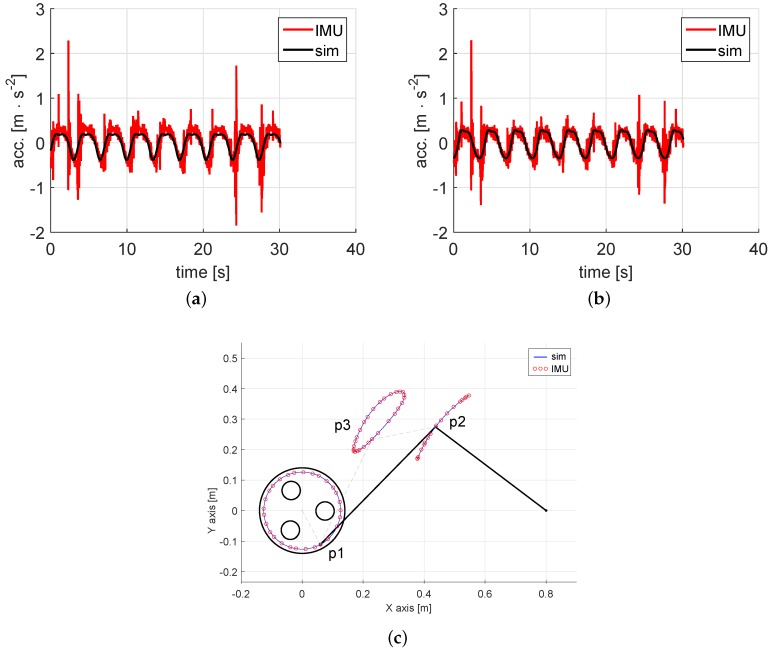
More experimental results: (**a**,**b**) acceleration measurements and predictions; (**c**) IMU-based and kinematics-based estimations of each interest point trajectory. (**a**) Acceleration of p3 in *x* axis; (**b**) Acceleration of p3 in *y* axis; (**c**) Trajectory of interest points $p_{1}$, $p_{2}$ and $p_{3}$.

It is well known that a constant velocity input in a four-bar mechanism produces variable accelerations in the coupler and rocker links. In this experiment, these accelerations are also measured by accelerometers inside the inertial units. Specifically, we are interested in the acceleration of point $p_{3}$, as seen in the global-reference framework. As shown in Equation (10), these variables are determined by transforming the signals, from the first IMU accelerometer local *x* and *z* axes, into the global *x* and *y* axes. Despite the noisy nature of this kind of sensor, the results show reasonably accuracy since the graphs depicted in Figure 6a,b fit the simulation results.

Finally, regarding the second goal of our experimental validation, Figure 6c shows the trajectory followed by the points of interest during the experiment. These trajectories are determined straightforwardly from the simulation by means of Algorithm 1. However, for the one based on the IMUs measurements, it is necessary to perform the calculations expressed in Equation (11). It is remarkable how the trajectories predicted by both methods fit each other.

### 5.2. Dynamics-Based Estimation

In this experiment, the linkage evolves from an initial state with a certain amount of potential energy before the crank is released, until reaching a state of rest due to the energy losses from friction. This friction is not modeled in the simulations. However, it will be demonstrated that the state observer may estimate the linkage position only by taking into account the angular speeds provided by the IMUs. The kinematic model has been experimentally validated in the previous subsection and, hence, for the sake of brevity, we present results exclusively on the crank angular position.

**Figure 7:**
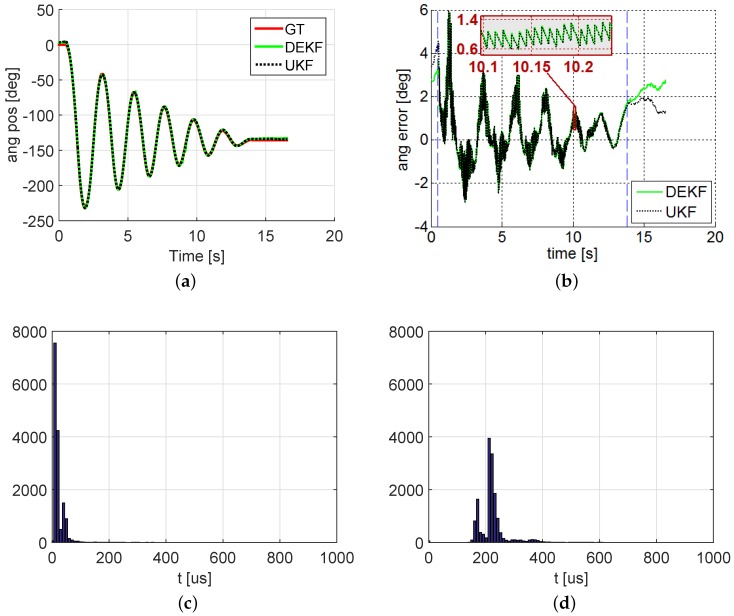
Results of the dynamic experiment: (**a**) Crank angle measured by the encoder and estimated by the Discrete Extended Kalman Filter (DEKF) and the unscented Kalman filter (UKF); (**b**) Estimation error of the crank angle; (**c**,**d**) Histogram of the computational cost of each filter. Due to the usage of non real-time operative systems, each timestep takes a different duration to complete. (**a**) Estimated and ground-truth crank angles; (**b**) Estimation error; (**c**) DEKF; (**d**) UKF.

In the case of the EKF, the matrix $\Sigma^{P}$ —which represents unmodeled forces and mechanical imperfections—has been tuned manually. For the computation of the covariance matrix of sensors noise $\Sigma^{S}$, the gyros standard deviation noise is measured and found to be 0.5 deg$\cdot s^{-1}$. The results of the state estimation are shown in Figure 7, in which the crank angle predicted by each filter is compared to the angle registered by the encoder attached to the crank. As can be seen, the results obtained with the two methods are almost indistinguishable in this example. The only significant differences are noted when the mechanism is not moving, whereupon the system becomes non-observable since the only sensor is a gyroscope. The valid section of the test is delimited by vertical dashed lines in Figure 7a,b. The RMSE found in this experiment in the valid region was 1.15 deg for the DEKF and 1.16 deg for the UKF, demonstrating that both methods can fuse information from the multibody model and gyroscopes to provide information on the position of the mechanism. When the mechanism is stopped, both methods become unreliable in a short time lapse.

Regarding the computational cost of each alternative, some consideration may be necessary. For this, the number of calls to the estimation functions and their performance has also been registered during the experiment. Thus, in Figure 7c,d, a summary a histogram of the of these function carried out by both filters is depicted. It can be seen that DEKF is significantly faster than UKF in its average computational cost. This fact must be taken into account when dealing with models with a larger number of states, where the UKF would be severely penalized. However, it is worth pointing out that, in both cases, the time consumed by the filters allows them to be run in real time for all time steps: we used a time step of $\Delta_{t}$ = 1 *ms*, while the average time spent by the filters was clearly shorter. Note that, due to the use of an operating system without a real-time kernel (Windows 7), the maximum duration of a time step cannot be *guaranteed*, in general, to be below $\Delta_{t}$ for all timesteps, although in these experiments both filters fulfill real-time performance for 100% of the timesteps, as can be seen in the histograms. Summarizing, in this problem, the accuracy of the two methods is almost identical. The advantage of the DEKF is its lower computational cost, while the UKF is easier to implement.

## 6. Conclusions

In this paper, we have presented different approaches for kinematic and dynamic simulation of multibody systems. Also, a method for estimating states under two scenarios has been proposed. This methodology has been applied to a well-known problem, *i.e.*, the four-bar linkage. In the first scenario, the value of the degree of freedom of the mechanism is defined and therefore it results in a kinematic problem. The second involves its equations of motion coupled with estimation techniques since only angular velocities from IMUs are provided. As a means of performing an experimental validation of these estimation algorithms, a testbed equipped with a series of sensors has been developed. The main contribution of this work consists of the experimental demonstration of the suitability of the DEKF and UKF for performing online estimations of the dynamic state of multibody systems. We show that, while the two filters used give good results in terms of accuracy, the DEKF has proved to be considerably faster in terms of computational efficiency. Future studies will examine the behavior of other filters found in the literature in combination with the use of different kinds of sensors as well as the experimentation with a three-dimensional testbed.

## Figures and Tables

**Table 1:** Notation summary for variables from the theoretical model (last column) and each sensor, *i.e.*, IMUs and optical encoder.

Variable	IMU 1	IMU 2	Enc 1	Model Coord.
Acceleration ($m\cdot s^{-2}$)	*acc_x_,acc_z_*	-	-	${\ddot{x}}_{3}$,${\ddot{y}}_{3}$
Pitch angle (rad)	*φ*	*ψ*	-	-
Pitch velocity ($rad\cdot s^{-1}$)	$\dot{\varphi }$	*$\dot{\psi }$*	-	-
Angular position (rad)	-	-	$\theta_{mot}$	$\theta_{1}$,$\theta_{2}$
Angular velocity ($rad\cdot s^{-1}$)	-	-	$\omega_{mot}$	$\dot{\theta_{1}}$,$\dot{\theta_{2}}$

**Table 2:** Notation summary.

Symbol	Description
z	Vector of independent coordinates
q=q(z)	Vector of dependent coordinates
Φ(q)=0	Constraint equations
$\Phi_{q}$	Jacobian of **Φ** with respect to **q**
M	Mass matrix
Q	Vector of generalized forces
$x,\hat{x}$	Real value and estimation of the filter state vector
${\hat{x}}_{k}^{-},{\hat{x}}_{k}^{+}$	Estimation mean at time step *k*, before and after the update stage
$P_{k}^{-},P_{k}^{+}$	Estimation covariance at time step *k*, before and after the update stage
$f(\cdot ),f_{x}$	Transition model and its Jacobian w.r.t. $\hat{x}$
$h(\cdot ),h_{x}$	Observation (sensor) model and its Jacobian w.r.t. $\hat{x}$
$o_{k}$	Sensor measurements at time step *k*
$\Sigma^{P}$	Covariance matrix of system transition ("plant") noise
$\Sigma^{S}$	Covariance matrix of sensors noise
K	Kalman gain matrix
$I_{N}$	The N×N unit matrix
